# Two simple and inexpensive methods for preparing DNA suitable for digital PCR from a small number of cells in 96‐well plates

**DOI:** 10.1002/jcla.23513

**Published:** 2020-08-05

**Authors:** Ziang Zou, Linna Guo, Parimah Ahmadi, Philip Hartjen, Martin Gosau, Ralf Smeets, Lan Kluwe

**Affiliations:** ^1^ Laboratory for Tumor Genetics and Regenerative Medicine Department of Neurology University Hospital Hamburg‐Eppendorf Hamburg Germany; ^2^ Department of Oral and Maxillofacial Surgery University Hospital Hamburg‐Eppendorf Hamburg Germany; ^3^ Department of Medicine University Medical Center Hamburg‐Eppendorf Hamburg Germany

**Keywords:** 96‐well plates, chelex100, digital PCR, direct PCR, small number of cells

## Abstract

**Background:**

Although DNA of high quality can be easily prepared from cultured cells with commercially available kits, many studies involve a large number of samples which increases the cost drastically. We optimized two simple and inexpensive methods for preparing DNA suitable for digital PCR from a small number of cells directly from wells of 96‐well plates.

**Methods:**

Cells (number: 10^3^‐10^4^) were lysed with a Direct PCR^®^ lysis buffer or a 10% Chelex100® solution. The lysates were further purified and concentrated by means of DNA precipitation with a blue‐colored glycogen as a carrier. PCR and digital PCR were used to evaluate the efficiency of the two methods.

**Results:**

For 1000 cells from one primary culture and two tumor cell lines, DNA was reproducible and obtained with recovery rate (obtained/expected amount of DNA) in the range of 50%‐90% as measured by the fluorometer dyes instrument Qubit. Using 8 out of a total of 10 µL DNA solution for 1000 cells, both conventional PCR and digital PCR were successful. For digital PCR, more than 1600 positive droplets were obtained for DNA from 1000 cells using the Direct PCR^®^ method, corresponding to a yield efficiency of approximately 80%. Further reducing the number of cells down to 100 would be possible with 160 positive droplets expected. Both reagents are inexpensive (0.08€/sample).

**Conclusions:**

Two methods are efficient, especially the Direct PCR^®^ reagent‐based method provides a simple and inexpensive method for preparing DNA suitable for digital PCR from small number of cells.

## Graphical abstract

With commercially available kits, DNA of high quality can be easily prepared from cultured cells. However, many studies involve a large number of samples which increases the cost drastically. In addition, a limited amount of each sample often is also a challenge. We optimized two simple and inexpensive methods for preparing DNA suitable for digital PCR from a small number of cells directly from wells of 96‐well plates. Using 8 out of the total of 10µl DNA solution for 1000 cells, both conventional PCR and digital PCR were successful. For digital PCR, more than 1600 positive droplets were obtained for DNA from 1000 cells using the Direct PCR® method, corresponding to a yield efficiency of approximately 80%.

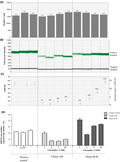

## 1 INTRODUCTION

With commercially available kits, DNA of high quality can be easily prepared from cultured cells. However, many studies involve a large number of samples which increases the cost drastically. In addition, a limited amount of each sample is often a challenge, for example, for studies using cells of primary cultures for testing multiple drugs at multiple concentrations in multiple replicates,^1^, ^2^, ^3^ and studies or diagnosis involving defined subpopulations of human immune cells.^4^ Therefore, simple and inexpensive methods for preparing DNA from a small number of cells are preferable.

Recently, we carried out a study for testing drug efficacy and specificity by treating the tumor and non‐tumor cells in a mixed culture. A tumor‐specific mutation (BRAF c.1799T>A) was quantified using digital PCR and DNA prepared from the cells treated with the drug at various concentrations in wells of a 96‐well plate. We used a Direct PCR reagent to lyse the cells directly in the wells and used 2 µL (out of a total of 50 µL) supernatant for digital PCR without further purification. The results of digital PCR were adequate at lower drug concentrations. However, at higher drug concentrations, the majority of the cells were destroyed, the number of positive droplets was too low (<20), resulting in high variability of the data.^5^ In order to increase the amount of DNA for digital PCR, we increased the lysate from 2 µL to 10 µL which led to complete failure of the droplet formation. This is likely due to an increased amount of detergent or other contaminants which are components of the Direct PCR reagent.

Therefore, we designed the present study to improve and optimize the Direct PCR method in order to obtain DNA suitable for digital PCR. We also included another method using Chelex100.^6^ We tested various cell numbers in each well (500 to 1 × 10^4^) and used a primary culture and two established cell lines as the cell sources. The prepared DNA samples were evaluated for their suitability for conventional PCR and for the droplet digital PCR.

## 2 MATERIALS AND METHODS

### 2.1 Cells prepared

**FIGURE 1:**
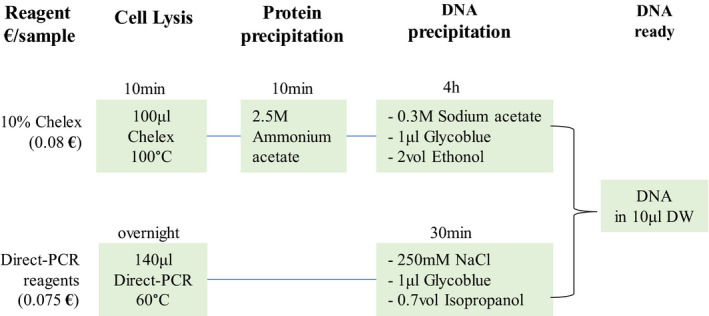
Schematic illustration of the two methods

Cells were used from a primary culture derived from a benign plexiform neurofibroma, a melanoma cell line A2058, and a human fibroblast line CCD18Co (both lines from ATCC). The cells were seeded into wells of 96‐well plates with at 500, 1 × 10^3^, 3 × 10^3^, 5 × 10^3^, 7 × 10^3^, and 1 × 10^4^ cells/well, each in three replicates. The plates were incubated overnight for the cells to attach the cultural surface and subjected to DNA extraction on the next day after checking the adhesive living cells under a microscope (Figure 1).

### 2.2 Lysis using a direct PCR^®^ lysis buffer

After removing the medium, the wells were washed twice with PBS. Subsequently, 70 μL of the Direct PCR^®^ lysis reagent (PeqLab, #Viag401‐E) supplemented with 0.2 mg/mL fresh proteinase K together with 70μl water was added to each well containing adhesive living cells. The solution in the wells was pipetted up and down several times using a multichannel pipet. The 96‐well plate was then placed on a heater at 60°C overnight to lyse the cells. On the next day, several wells were checked under a microscope to ensure complete lysis. The plates were then heated at 85°C for 45 minutes on the heater to inactivate the proteinase K. Afterward, the plates were loaded into a centrifuge to spin down the debris at 250 *g* for 1‐2 minute. Supernatants containing the extracted DNA were further purified by precipitation.

### 2.3 **Lysis using chelex100** ^®^

The chelex100^®^ powder (BioRad, #142‐1253) was dissolved in water at 10% and heated to 100°C. To each well of a 96‐plate, 200 μL of the 10% chelex100 solution was added and the plate was heated at 100°C on a heater for 10 minutes. The suspension in the wells was pipetted up and down several times using a multichannel pipet to enhance the lysis. The complete lysis of the cells was checked under a microscope. The plate was then centrifuged at 1750 *g* at 4°C for 1 minute. The volume of the supernatant was measured and ½ volume of 7.5 mol/L ammonium acetate was added to give a final concentration of 2.5 mol/L to precipitate the proteins. The suspension was vortexed until a yellow‐white protein precipitate appeared, kept on ice for 5 minutes to fix the precipitate complex and span down at 15 000 *g* at 4°C for 10 minutes. The supernatant containing DNA was subjected to the subsequent precipitation.

### 2.4 DNA precipitation

To eliminate contaminants such as detergents and salts from the lysates, a DNA precipitation step was added to both methods. The lysates were transferred into 0.5 mL tubes and to each tube, sodium acetate or NaCl were added to final concentration of 0.3 or 0.25 mol/L, respectively. As a carrier for low concentration DNA, 1 µL Glycoblue Coprecipitant (Thermo Fisher # AM9515) was added. This is a blue‐colored glycogen for better visualization of the precipitation pellet. Finally, 2 volumes of ice‐cold ethanol or 0.7 volume of isopropanol were added. To enhance DNA precipitation, the mixtures were kept in −20°C freezer for at least 4 hours before the centrifugation at 15 000 *g* and 4°C for 20 minutes. Blue precipitates were visible at the bottom of the tubes which were then washed once with 75% ice‐cold ethanol or 100% ice‐cold isopropanol. After drying in a culture hood, the pellets were dissolved in 10 µL water/sample at 55°C for 5 minutes and quantified using 2 µL with Quant‐iT™ BRDNA Assay Kit (QuantiT™ PicoGreen^®^, Invitrogen) on a Qubit^®^ Fluorometer dyes instrument.

DNA yield of the two methods was compared using a *t* test with two‐tailed hypothesis. The significant level was set at 0.05.

### 2.5 PCR and digital PCR

Conventional PCR was carried out using 2 out of the 10 µL DNA and a primer pair for an exon of the *NF1* gene which is used for the routine genetic diagnosis in our laboratory. The amplification was carried out for 35 cycles at annealing temperature of 60°C. For each setting, a negative control containing no DNA and a positive control containing high‐quality DNA were included.

Digital PCR was carried out using the rest 8 µL DNA with an assay kit for the *RPP30* gene (BioRad, #dHsaCP2500350) on a BioRad system QX100.^3^

## 3 RESULTS

**FIGURE 2:**
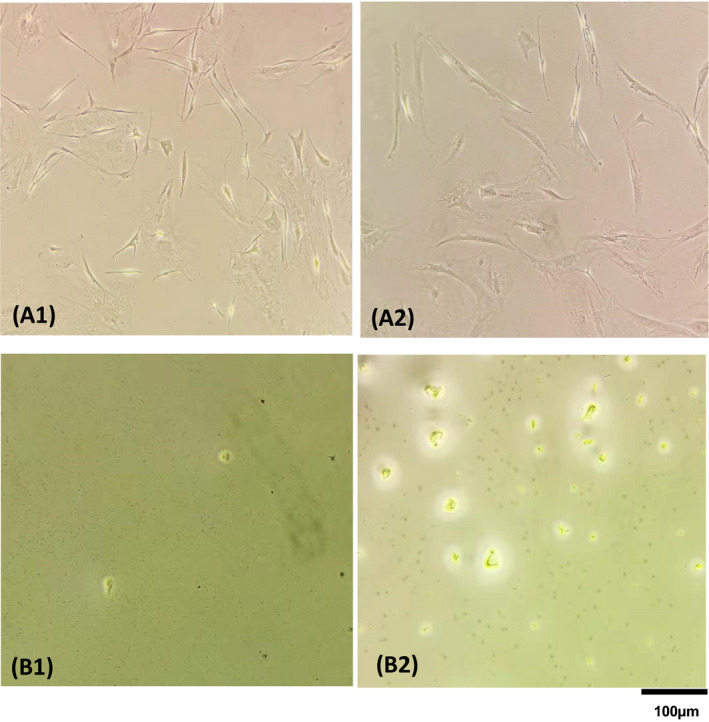
Cells in wells of a 96 plate before (A) and after (B) lysis cell by Direct PCR (B1) and Chelex100 (B2)

The procedures using the Direct PCR lysis reagent and the 10% chelex100 were illustrated in Figure 1. After the lysis, no living cells were visible anymore indicating complete lysis (Figure 2).

**FIGURE 3:**
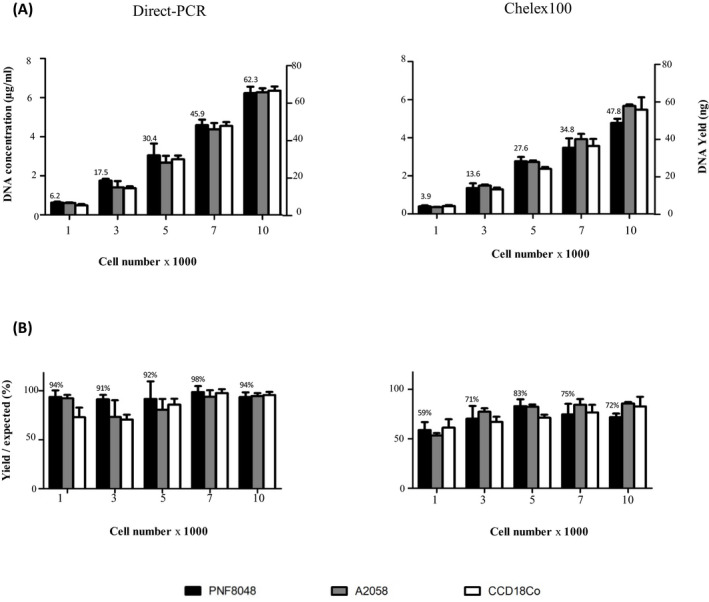
DNA concentration measured using the Quant‐iT™ BRDNA Assay Kit on Qubit^®^ Fluorometer (A, left scale), calculated total yield of DNA (A, right scale) and calculated DNA recovery rate (B)

DNA was measurable on Qubit^®^, a Fluorometer dyes instrument, for 10^3^ cells and more. The DNA yield increased with the number of cells linearly (Figure 3A), and the recovery rate (yield/expected) did not change much for different types and numbers of cells (Figure 3B). The yield of the Direct PCR method was significantly higher than that of the chelex method for 1000, 7000, and 10 000 cells (*P* < .05). Due to the high variation in DNA prepared from 3000 and 5000 cells, there was no statistical difference between the yield of these two methods. DNA from <1000 cells was not measurable using a fluorometer dyes instrument.

**FIGURE 4:**
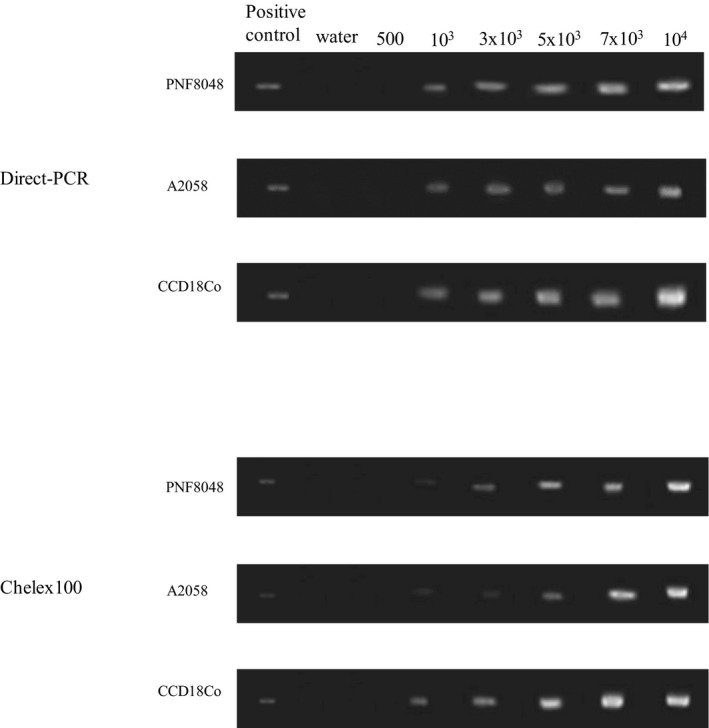
PCR products were visible on agarose gels for DNA from 1000 or more cells. Positive controls are DNA prepared using a conventional method from human leukocytes. Water was used as the negative control to exclude contamination. The primary culture and the two cell lines as the source were given on the left. The DNA preparation methods are indicated on the most left

In concordance, PCR products were visible on agarose gels for DNA from 1000 cells and more with 35 amplification cycles (Figure 4). By contrast, DNA from 500 cells was not sufficient for giving a visible PCR even after 40 cycles (data not shown). For all PCR, no amplification was visible for the negative control of water, excluding the possibility of non‐specific amplification.

**FIGURE 5:**
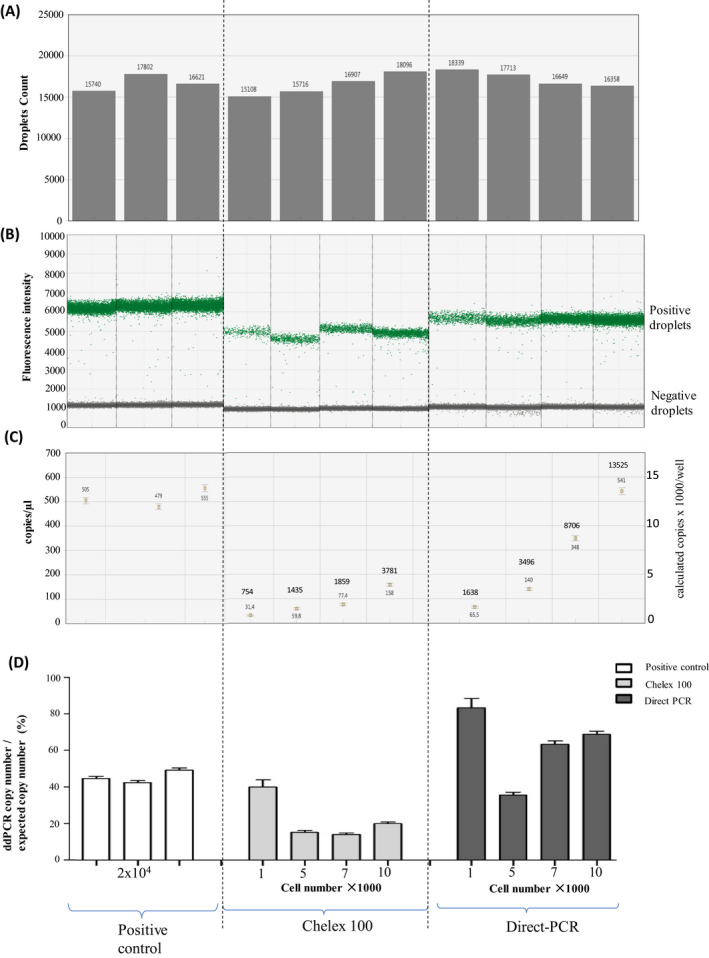
Digital PCR amplifying a RPP fragment. A, >15 000 total droplets were reached for all samples; B, clear separation of the positive and negative droplets in the fluorescence intensity. C, copy numbers/µL from digital PCR QX100 software (left scale) and calculated numbers of copies in each well (right scale and at each data point). D, calculated DNA extraction efficiency based copy number from digital PCR

Digital PCR was successful for DNA from 1000 cells (Figure 5). More than 15 000 droplets were obtained for all samples (Figure 5A). More 1600 positive droplets were obtained for DNA from 1000 cells using the Direct PCR methods (Figure 5C), corresponding to a recovery rate of approximately 80% (Figure 5D). Less copy number (<900) was obtained for DNA from 1000 cells prepared using the chelex method.

## 4 DISCUSSION

We optimized two simple and inexpensive methods for preparing DNA directly from 1000 cells in wells of 96‐plates and demonstrated their suitability for PCR and digital PCR. No clear difference was seen among the three different cultures used as the cell source, indicating that the methods are applicable for any type of cells.

The Direct PCR reagent method is clearly more advantageous. It does not have the protein precipitation step and therefore is simpler than the Chelex100 method. More importantly, the DNA copies yield reached 80% of the expected one from 1000 cells. According to the Quibit measurement, the recovery rate reached 90%. However, there is likely some overestimation since the cells were cultured overnight for attachment and some growth is possible. Presuming a 20% cell growth, the DNA yield would decrease to 75% and 67% for the Quibit measurement and the digital PCR, respectively, which we believe are more realistic.

With its special formulation, the Direct PCR reagent lyses the cells overnight to release DNA. After inactivating the proteinase K, the crude supernatant can be directly used for PCR. A small amount (1/25) of the supernatant can also be used for digital PCR.^5^ However, increasing the amount of crude lysate resulted in a failure of droplet formation, likely due to the too high concentration of detergent and/or other inhibitory components. Since the composition of the Direct PCR reagent is not disclosed by the supplier, the exact cause for the failure of droplet formation is unknown. In any case, we demonstrated that precipitation is sufficient to solve this problem. In addition, precipitation enables concentrating the DNA so that the entire DNA from one sample can be used for one digital PCR measurement. In the present study, >600 positive droplets were obtained from 8/10 of DNA from 1000 cells. With the limit of detection in one digital PCR measurement as 4 copies,^7^ the cell number per sample can be theoretically further reduced to <10.

Chelex100 is a detergent that breaks the cell membrane and releases DNA.^8^, ^9^, ^10^ Compared to the Direct PCR method, an additional protein precipitation step is necessary. The DNA recovery of approximately 50% is only about half of that of the Direct PCR method, likely due to DNA loss in the protein precipitation process. Future studies should address the issue if this precipitation can be omitted without reducing the quality of the DNA.

Using glycogen as a carrier, a high recovery rate (75%) was achieved also for small amount of DNA from 1000 cells. In addition to eliminating inhibitory component, precipitation also enabled reducing the volume of the DNA. To visualize the DNA‐glycogen pellets and reduce the risk of losing them, we used a blue‐colored glycogen in the present study. A disadvantage of the Blueglycogen is the relatively high cost which is approximately 0.2€/sample.

By contrast, the costs for the Direct PCR lysis reagent and the Chelex100 resin are both low with approximately 0.08€/sample. One possibility to further reduce the total cost is to replace the colored glycogen with uncolored glycogen, reducing the costs by approximately ¾. The additional costs for other reagents such as sodium acetate and ethanol are only marginal. Altogether, the cost for one sample will be 0.15‐0.3€, depending on using colored or uncolored glycogen. In comparison, a commercial kit costs more than 1€/sample. In addition, the methods in the present study can be applied to various numbers of the sample in a 96‐plate and do not require special devices.

In summary, we optimized two methods for preparing DNA with the following features and advantages:

1. Simple, quick, easy, and reliable
2. Substantially lower cost than the commercial kits
3. Optimized for small number of cells and samples in wells of 96‐plates
4. Satisfactory yield (75%)
5. Quality of the DNA suitable for conventional and digital PCR.
